# Can the Establishment of National Key Ecological Function Areas Enhance Vegetation Carbon Sink? A Quasi-Natural Experiment Evidence from China

**DOI:** 10.3390/ijerph191912215

**Published:** 2022-09-26

**Authors:** Tongyue Zhang, Mengyang Hou, Liqi Chu, Lili Wang

**Affiliations:** 1 College of Economics and Management, Northwest A&F University, Yangling, Xianyang 712100, China; 2 School of Economics, Hebei University, Baoding 071000, China; 3 Center of Resources Utilization and Environmental Conservation, Hebei University, Baoding 071000, China

**Keywords:** National Key Ecological Function Areas (NKEFAs), vegetation carbon sink, time-varying DID, heterogeneous

## Abstract

The National Key Ecological Functional Areas (NKEFAs) of China rely on the main functional area planning, with the core goal of enhancing the supply of ecological products. Carbon sink is an important ecological product, and it is necessary to understand whether the establishment of NKEFAs has enhanced vegetation carbon sink (CS). Considering the establishment of NKEFAs as a quasi-natural experiment, based on the panel data of prefecture-level cities in China from 2001 to 2019, a time-varying difference-in-differences (DID) model is used to systematically examine the impact of NKEFAs on CS. The study found that the establishment of NKEFAs has significantly enhanced the CS, and compared to the non-NKEFAs, NKEFAs has increased CS in the covered areas by an average treatment effect (ATE) of 2.1625. The establishment of NKEFAs can enhance CS through the optimization of territory spatial structure, the upgrading of industrial structure and the inter-industrial mobility of labor. The enhancement roles of NKEFAs on CS are heterogeneous across different functional area types, geospatial locations, and quantile levels, with higher enhancement of CS at windbreak–sand fixation type, northwestern region and high quantiles, respectively. In addition, NKEFAs not only have a significant positive ecological spillover effect, but also balanced with local economic growth, they achieve the goals of “lucid waters and lush mountains are invaluable assets”.

## 1. Introduction

China has entered a new normal of high-quality socio-economic transformation and development. Facing the threat of climate change and environmental problems, the Chinese government has made a commitment to reach peak carbon emissions by 2030 and achieve carbon neutrality by 2060 [1]. Then, the realization of the dual-carbon strategy and the improvement of ecological environment are the basic of high-quality and green development. The territory is formed by the interaction of terrestrial ecosystems and human activities [2]. The degradation of terrestrial ecosystems in the land space due to inappropriate practices and the unreasonable structure of human activities is an important reason for the increase in carbon emission intensity and environmental problems [3], which is highlighted by the structural contradictions and conflicts in the utilization of land space caused by the rough expansion of industrialization and urbanization, and the production and living space squeezes the ecological space in an all-round way, resulting in ecosystem degradation, prominent environmental problems and irrational territory structure [4].

To rationally develop and utilize the territory, China issued the “National Main Function Area Planning” in December 2010, which clarifies the main functions of different regions and gradually forms a land territorial spatial development pattern that integrated population, economy, resources, and the environment [5]. According to the development mode, the territory can be divided into four functional areas: optimized development, key development, restricted development, and prohibited development. According to the development content, the main functional areas can be divided into urbanized areas, main agricultural products-producing areas and key ecological functional areas. Among them, the National Key Ecological Function Areas (NKEFAs) take protecting and restoring the ecological environment and enhancing the production capacity of ecological products as the primary tasks, which are positioned as important areas to ensure national ecological security, and undertaking important ecological functions such as water conservation, soil conservation, windbreak sand fixation and biodiversity maintenance, as the NKEFAs provide an important carbon bank and ecological barrier in China. The NKEFAs minimize interference with the natural ecosystem by strictly limiting different kinds of development activities and implementing strict environmental standards for industrial access. In addition, suitable industrial development and infrastructure construction should be controlled within the smallest possible spatial scope, and the ecological space area should be guaranteed not to be reduced, so as to promote the rational layout of the territory space and guide the layout of population and industry to be compatible with the carrying capacity of the resources and environment.

Currently, at the end of 2010, China established the first list of 25 NKEFAs with restricted development, covering 436 county-level administrative regions [6], and it added 240 new counties into the NKEFAs in 2016 (Appendix A). Since the beginning of 2010, the NKEFAs have become the unique and largest existing regional ecological compensation policy in China [7]. In addition, the distribution area of NKEFAs is an important natural ecological barrier in China, and it has a high supply capacity of ecological products, which plays an important role in neutralizing carbon emissions and improving the ecological environment. Carbon sink (CS), as an important ecological product [8], reflects the capacity and scale of vegetation to absorb and store carbon dioxide, and its main absorption processes include the photosynthesis of plants and carbon capture, utilization, and storage (CCUS) technology [9]. So, under the background of the dual carbon strategy of “carbon reduction and carbon neutrality”, has the establishment of NKEFAs effectively enhanced CS? This is an important question that deserves attention and in-depth discussion. Thus, fully examining and objectively assessing the impact of NKEFAs on CS is not only conducive to the accumulation of experience and institutional improvement in the sustainable implementation of ecological policies but also provides useful reference for promoting the realization of dual-carbon goals and the balanced development of ecological protection and high-quality economy.

## 2. Literature Review and Theoretical Analysis

The NKEFAs, as a specific area in the main function area system that undertakes ecological functions and maintains ecological security, function as the location-oriented ecological compensation policy implemented in China [10], which also can be regarded as a command-and-control environmental regulation [11]. Then, investigating the CS generated by the NKEFAs belongs to the typical category of policy effect assessment. At the present stage, for the ecological effects of NKEFAs, relevant studies mainly include the effect evaluation of the ecological transfer payment system and the quantitative elaboration of ecological benefits such as ecosystem services in the NKEFAs.

Under the current system, NKEFAs generate positive incentives for local governments mainly through ecological transfer payments, which in essence reflect the principal–agent relationship between the central government and local governments. The central government as a principal and the local government as an agent sign a long-term transfer payment contract for ecological compensation, which motivates local governments to invest more efforts in ecological protection [12] and to enhance the financial capacity of the governments of NKEFAs to provide basic public services [13]. In terms of the ecological effect of the ecological transfer payment system in China, Li Guoping conducted rich research from the perspectives of policy interpretation, incentive effect, fund allocation, compensation standard, etc. His early research found that the ecological compensation effect of NKEFAs is not significant, which is closely related to the implementation of the national key ecological function area transfer payment policy [14]. On the one hand, the transfer payment funds for NKEFAs are not tilted toward the NKEFAs with weaker fiscal resource and poorer ecological environment quality, resulting in a lack of efficiency and targeting of funds use [15]; on the other hand, both the central and local transfer payment schemes for NKEFAs suffer from the mismatch between the dual objectives of protecting the ecological environment and improving people’s livelihood and the performance assessment index system [16].

Most of the studies on the evaluation of ecological transfer payment effects belong to experience summarization and theoretical discussion, and quantitative analysis mostly focuses on case studies. Li et al. [17] conducted research on the Qinba Mountains in Shaanxi and found that although the transfer payment for NKEFAs promoted the improvement of environment quality, the promotion effect is relatively weak. In contrast, Xu et al. [18] found that the positive ecological protection effect of transfer payment for NKEFAs in Shaanxi Province was relatively significant, and the ecological environment in the base period has played an indispensable role in promoting it. The reason for the different ecological effects may be due to the earlier time of Li’s empirical test and the short time interval, which makes it difficult for the policy to be effective in a short period of time. In addition, Miao and Zhao [19] found that the transfer payments for key ecological function areas generally improved the quality of ecological environment represented by water quality, and the improvement effect was significantly dependent on the local government’s environmental protection expenditure efforts, but the ecological transfer payments only played a financial compensation effect and did not play the proper institutional incentive function. Zhu and Chen found that the transfer payment for key ecological functional areas effectively improved the ecological environment of Guangdong Province, and the improvement effect showed an increasing trend [7].

Considering the ecological effects on NKEFAs, there are also studies comparing the policy before and after the implementation through remote sensing technology and GIS technology, both of which found that the ecological environment of NKEFAs generally showed an improvement trend [20], but there were differences in the improvement of ecological conditions in different types of function areas [21]. Some studies found that the ecosystem quality from 2000 to 2010 before the establishment of NKEFAs improved, but the improvement was less than that of non-NKEFAs [22], and the overall ecosystem service value of NKEFAs showed a significant increase after 2010, but the ecosystem service value of different functional types is soil conservation type > biodiversity maintenance type > water conservation type > windbreak sand-fixation type [23]. In general, existing studies have focused more on the environmental improvement effect of NKEFAs; however, the other major goal of NKEFAs—enhancing the supply capacity of ecological products—lacks the necessary attention. Thus, since carbon sink is an important ecological function and product, it is necessary to pay attention to the CS of NKEFAs.

NKEFAs focus on enhancing the service function of ecological products. In theory, the protection and restoration of ecological environment in the NKEFAs can help to improve vegetation carbon sink in several ways.

**Figure 1:**
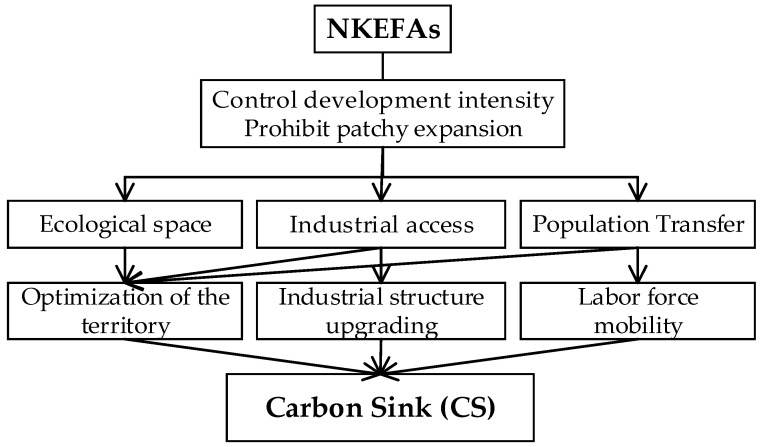
The path of NKEFAs affecting CS.

(1) NKEFAs can optimize the pattern of territory development. The NKEFAs expand ecological space, clearly restrict large-scale and high-intensity urbanization and industrialization, and strictly control the development intensity and scope in the territory development according to the carrying capacity of regional resources and environment [24]. Urban construction and industrial development should be concentrated, and stronghold-type development in existing towns with relatively strong carrying capacity of resources and environment requires the full delineation of ecological red lines. The delineation of restricted development areas and ecological red lines promotes the optimization of territorial spatial development pattern, improves the efficiency of land spatial allocation [25], and maintains ecological function while minimizing restrictions on human land use [26].
(2) NKEFAs can promote industrial structure upgrading. The negative list of industrial access in NKEFAs clarifies the list of industries restricted and prohibited from development, implements targeted industrial access and environment access policies and standards, supports the appropriate utilization of special resources, and rationally develops suitable industries. For existing industries that are not suitable for the main function positioning, it will create a crowding-out effect on polluting enterprises [27,28], promote industrial gradient transfer or elimination, and production factors will gradually transfer to the service industry. When industrial policies become stricter, local governments have more incentives to promote the upgrading of industrial structure, eliminate outdated production capacity, and guide the development of less polluting suitable industries, special industries, and service industries such as tourism and sightseeing [29], triggering the inter-industrial flow of production factors and promoting industrial structure upgrading. The upgrading of industrial structure is conducive to the reduction in pollutant emissions, and the accompanying technological upgrade also reduces the constraints of resources and the environmental impact on economic development [30].
(3) NKEFAs can promote labor transfer and mobility. The limitations of large-scale urbanization development and industrial structure upgrading make it difficult for the NKEFAs to carry a larger population, and a part of the population will actively transfer to urbanized areas with more employment opportunities. Territory spatial development will also lead to an orderly transfer of population from restricted development areas to key development areas, and urbanized areas will increase the corresponding labor force to ease employment pressure and increase population density in built-up areas [31]. The population is concentrated on a large scale within the spatial unit of environmental capacity, which promotes the reallocation of factors and resources, and it facilitates the prevention of environmental pollution and the effective use of resources, thereby enhancing and improving the supply of ecological products (Figure 1).

Based on the above literature review and theoretical analysis, this paper focuses on the impact of NKEFAs on CS. Specifically, the NKEFAs is regarded as a “quasi-natural experiment”, and the panel data of 330 cities at the prefecture level and above in China from 2001 to 2019 are used as research samples. A time-varying difference in difference (DID) model is used to assess the impact of NKEFAs on CS. In addition, the event-study approach (ESA) is used to examined the parallel trend and dynamic effects of this policy, and we conduct a series of robustness tests such as placebo test, excluding the influence of other policies. The heterogeneity impact of this ecological policy in terms of functional area type, geospatial distribution, and different quantile levels is further discussed. This paper also expands the analysis to examine whether there is a spatial spillover effect in the impact of NKEFAs on CS, and it examines whether ecological objectives can be balanced with economic growth.

The marginal contributions of this paper are mainly reflected in the research scale, research content, research method and research depth: (1) Research scale. The research samples of the existing literature are mainly provincial panel data or case study of a certain province, which has limited coverage, and the inter-provincial average treatment effect can hardly reflect the individual variability of ecological characteristics within the province accurately. In this paper, a more detailed observation will be made at a smaller scale at the prefecture level [32] to overcome the homogenization error of provincial macro data to a certain extent. (2) Research content. Most of the existing studies only focus on the ecological effect or carbon emission reduction in NKEFAs. One of the goals of NKEFAs is to improve the supply capacity of ecological products, but carbon sequestration, as an important ecological product, has received little attention. (3) Research method. The existing studies using GIS technology focus on a comparative analysis of changes in the ecological environment before and after the establishment of NKEFAs or between national key and non-key ecological function areas, but it is difficult to remove the influence of other ecological policies by the interpretation of remote sensing images, and identification using the DID approach can effectively strip out the impact of other ecological policies. (4) Research depth. The analysis of heterogeneity and mechanism in existing studies is still insufficient, and the in-depth examination of the heterogeneity and mechanism of this ecological policy in this paper can enrich the study of policy causality identification.

## 3. Materials and Methods

### 3.1. Time-Varying DID Model

Among the methods used to assess the effects of policy implementation, the DID model (difference in difference) is an econometric method that has been widely used in recent years. The basic idea of this method is to consider the implementation of a new policy as a “quasi-natural experiment” that is exogenous to the economic system [33], which has the advantage of effectively stripping out the net policy effects of a specific policy and overcoming potential endogeneity problems [34]. The NKEFAs may make the CS in covered areas differ before and after the policy implementation on the one hand and between covered and non-covered areas on the other. The model regression design based on these dual differences can effectively control the effects of other co-occurring policies and the ex ante differences between covered and non-covered areas, and then, it can identify the net effect of policy shocks affecting CS. Therefore, these dual differences allow this study to consider the establishment of NKEFAs as a “quasi-natural experiment” and to assess its policy effect using a DID model.

The “National Main Functional Area Planning” was introduced at the end of December 2010, which announced the first list of counties with NEKEFAs at the same time, and the list of additional counties was announced in 2016. The main ecological objectives of the two batches of covered counties are basically the same, i.e., to enhance the supply capacity of ecological products, one of which is the improvement of CS capacity. If the establishment of NKEFAs achieves the expected main objectives, it shows that the ecological policy helps to protect and restore the ecological environment and promote the process of carbon neutrality.

There are currently two batches of NKEFAs established, and the timing of policy shocks differs in different regions, which is suitable for assessment using the time-varying DID model [35]; then, the model is constructed as follows:


(1) $$Y_{it}=\alpha +\beta DID_{it}+\delta control_{it}+\mu_{i}+\gamma_{t}+\varepsilon_{it}$$

In Equation (1), *Y_it_* denotes the explained variable (CS), *DID_it_* denotes the core explanatory variable, *DID_it_* = *treat_i_* × *post_t_*, *treat_i_* denotes the grouping dummy variable, *post_t_* denotes the time dummy variable, *β* reflects the net policy effect of NKEFAs, if *β* > 0, the establishment of NKEFAs helps to enhance CS. *control_it_* denotes the set of control variables, *μ_i_* denotes the area-fixed effect, *γ_t_* denotes the time-fixed effect, *ε_it_* is the random disturbance term.

The rule for *treat_i_* is: when region *i* belongs to the NKEFA in year *t*, *treat_i_* = 1; otherwise, *treat_i_* = 0. Since the list published in the policy document is based on counties, and the research scale of this paper is at the prefecture-level city, this paper will set the prefecture-level city where the counties in the list belong to the NKEFAs, and it will aggregate the county-level list to the prefecture-level city.

The rule for *post_t_* is: Since the time point for the establishment of the first list of NKEFAs is the end of 2010, and the time point for the added list is 2016, we take 2011 as the starting time for the treatment group, and when region *i* belongs to the first list, *post_t_* = 1 for the region in 2011 and after, *post_t_* = 0 before 2011, and for the added region i, *post_t_* = 1 for the region in 2016 and after, *post_t_* = 0 before 2016. Assuming that a prefecture has counties within its jurisdiction in both the first batch and the added list, we identify the policy time point of the prefecture as 2011.

### 3.2. Parallel Trend Test and Dynamic Effect

The DID method needs to satisfy the parallel trend assumption; that is, before the establishment of the NKEFAs, the trends of environmental effect changes in the treatment group and the control group are basically the same. The event-study approach (ESA) can not only observe the dynamic effect and persistence of policy impact but also make a parallel trend assumption so as to accurately determine whether there is a significant difference in the trend of change between the treatment group and control group in the NKEFAs [36]. So, we test the parallel trend assumption and analyze the policy dynamic effect based on the ESA [37].

Relying on the main functional area planning, the NKEFAs were officially implemented in 2011, and the second batch was added in 2016. Considering that the Ministry of Finance has promulgated the “National Key Ecological Function Areas Transfer Payment (Pilot) Measures” in 2009, and the ecological transfer payment is piloted in a small number of important ecological regions, the parallel trend test in this paper is conducted using the year before the implementation of NKEFAs (2010) as the reference group. Referring to related studies [38,39], Equation (1) was expanded as:


(2) $$Y_{it}=\alpha_{0}+\beta_{t}\sum_{t=2001,t\neq 2010}^{2019}treat_{i}\times year_{t}+\mu_{i}+\gamma_{t}+\varepsilon_{it}$$

In Equation (2), *treat_i_* × *year_t_* is the multiplication term of the grouping variable *treat_i_* and the year dummy variable *year_t_*, which is not introduced in the year before the policy implementation (2010) as the reference group. *β_t_* denotes the policy effect in each year; the change of this coefficient after 2010 can reflect the persistent impact of the policy. If *β_t_* is basically insignificant before 2010, the parallel trend assumption is satisfied.

### 3.3. Variable Selection

(1) Explained variables: carbon sink (CS). The scale of vegetation CS, which is mainly calculated from the net primary productivity (NPP) of vegetation, can reflect the supply capacity of ecological products. Specifically, CS is a process, activity or mechanism that absorbs CO_2_ from the atmosphere, such as plant photosynthesis [8], while NPP refers to the residual of gross primary productivity (GPP) after deducting the value of respiration of autotrophs (RA), which can be deduced from the CO_2_ absorbed and the dry matter produced by plant photosynthesis [40], and the chemical equation is 6CO_2_ + 6H_2_O→C_6_H_12_O_6_ + 6O_2_. Vegetation can fix 1.63 kg CO_2_ per for every 1 kg of dry matter produced, and the carbon content in dry matter accounts for about 45% of the total NPP, so the CO_2_ that vegetation can fix per unit area is W_CO2_ = NPP/0.45 × 1.63, its unit is g/m^2^, and then, it is multiplied by the area covered by vegetation to obtain the scale of CS [41].
(2) Core explanatory variable: NKEFAs
  ① The scope of the prefecture-level city (treat). According to the policy document, the first batch covers 436 county-level administrative regions, and the new list covers 240 county-level administrative regions. Since the study scale is prefecture-level cities, if a prefecture-level city jurisdiction covers a county in the list, it will be set as the treatment group and vice versa as the control group, and this policy is finally determined to cover 171 prefecture-level cities (the first batch of 111, the new additional of 60).
  ② The time node of policy implementation. According to the promulgation time of the “Main Functional Area Planning” and the time of the new list, it is determined that 2011 is the starting time of the first batch of NKEFAs, and 2016 is the starting time of the new list (approved by the Stata Council in September 2016).
(3) Control variables

The CS are also influenced by various factors such as socio-economic, basic factor endowment and natural climate, and these exogenous factors need to be controlled. They mainly include the following. Population density (DEN) reflects the growth in population size. Economic growth is represented by GDP per capita (PGDP), and we take it in its logarithmic form to reflect the overall situation of economic development. The urbanization rate (URBAN) reflects the expansion of urbanization. Industrial structure adjustment (STURC) reflects the changes in the proportion of the three industries in the national economy. The widening of the urban–rural income gap (GAP) attracts surplus rural labor to the cities and towns. Opening to the outside world (OPEN) reflects the effect of international trade liberalization on ecological improvement. Transportation accessibility (TRANS) is used to reflect the improved transportation infrastructure that accelerates the flow of factors and decreases the cost of communication. Natural climatic factors are mainly selected as precipitation (PRE), temperature (TEM), and sunshine hours (SUN) to control the interference of climatic conditions on CS changes.

### 3.4. Data Sources

The research sample in this paper is the panel of 330 prefecture-level and above cities in China from 2001 to 2019. The sample data of the treatment groups of NKEFAs are manually summarized and collated according to the Main Function Area Planning and the relevant documents of the new list. The NPP data required for CS calculation come from the MOD17A3HGF product based on the MODIS satellite released by NASA, with a spatial resolution of 500 m. The land use data are derived from the global land cover product data (www.esa-landcover-cci.org) of the European Space Agency’s Climate Change Initiative (CCI) at annual and 300 m × 300 m spatial–temporal resolutions, respectively. The data of climate variables such as temperature, precipitation and sunshine hours come from the annual data set of China’s surface climate data from the China Meteorological Data Network (www.data.cma.cn, accessed on 1 May 2021). The socio-economic data involved are mainly obtained from the China City Statistical Yearbook, China Regional Economic Statistical Yearbook and provincial statistical yearbooks in previous years, and the missing data are supplemented by prefecture-level city statistical yearbooks and statistical bulletins, and the still missing data are supplemented by interpolation. The variable description and descriptive statistics are shown in Table 1. The variance inflation factor (VIF) of each variable was significantly less than 10, and the average VIF was 2.14, indicating that there was no obvious multicollinearity problem among the variables.

## 4. Results and Analysis

### 4.1. Baseline Regression Result

Table 2 reports the baseline regression results of NKEFAs affecting CS, and column (1) controls for neither fixed effects nor variables, column (2) controls for fixed effects only, column (3) controls for variables only, and column (4) controls for both fixed effects and variables. The estimated coefficients of *DID* are all significantly positive, and there is a significant positive effect of NKEFAs on CS regardless of whether the year and city are fixed or not, and regardless of whether a series of socio-economic and natural characteristics variables are controlled. From the estimated DID coefficient in (4), the NKEFAs significantly enhance the CS of covered cities. Compared with cities in non-NKEFAs, NKEFAs have enhanced by about 2.1624 at the prefecture level in the average treatment effect (ATE). Thus, the question raised in the title can be tentatively answered, and the establishment of NKEFAs enhances CS, which is basically in line with its original intention and ecological goal of providing ecological products and services.

Among the control variables, the estimated coefficients of lnPGDP and PRE are positive significantly, which indicates that the higher level of regional economic development and sufficient precipitation conditions are conducive to the increase in CS. The estimated coefficients of DEN, URBAN, OPEN, TRANS, TEM and SUN are significantly negative, which indicates that increasing population density, higher urbanization level, higher level of opening to the outside world and transportation infrastructure, and higher temperature and sufficient sunshine will inhibit the increase in vegetation CS. The estimated coefficients of STURC and GAP are not significant, and the effects of industrial structure adjustment and urban–rural income gap on CS have not been revealed yet and need to be explored in depth.

### 4.2. Parallel Trend Test and Policy Dynamic Effect

**Figure 2:**
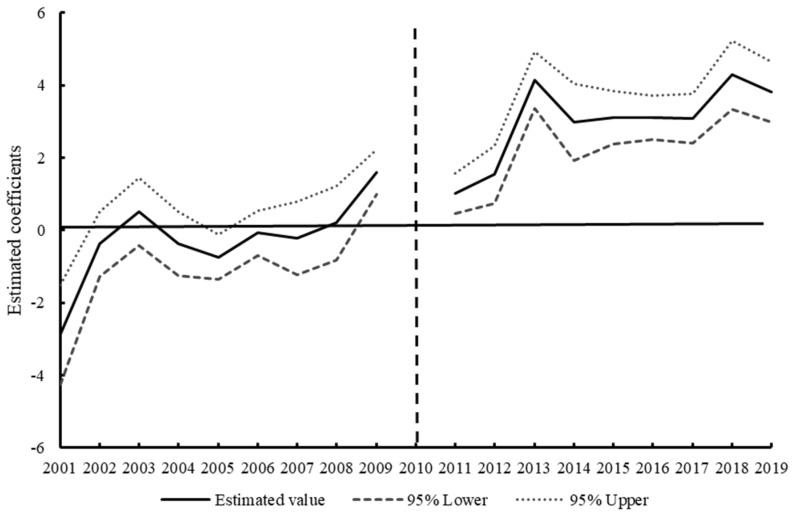
Dynamic effects of national key ecological function areas on CS.

The results show (Figure 2) that the estimated coefficients of the NKEFAs are basically insignificant and fluctuated around 0 for each year before establishment, indicating that the CS was not significantly different between the treatment group and control group before establishment, while the estimated coefficients in the year of implementation and subsequent years basically passed the test at the 1% significance level, so the parallel trend hypothesis of DID was satisfied.

In terms of dynamic effect changes, the estimated coefficients before the establishment of NKEFAs change from insignificantly negative to significantly positive, gradually showing a certain positive environmental effect, and the coefficient of NKEFAs on CS has changed to significantly positive in 2009, indicating that there is an expected effect of policy implementation on the impact of CS; relevant government departments can respond in advance according to the policy guidance. The possible reason is that in 2009, the state has begun to carry out the pilot project of ecological transfer payment in a small number of important ecological regions to strengthen ecological protection.

After the establishment of NKEFAs, the impact on CS is significantly positive in the average treatment effect (ATE), and this positive effect shows a dynamic upward trend, and there is continuity in time. The CS effect of this policy began to stabilize after 2 years after its establishment, and the obvious growth process after 2017 was due to adding a second batch of lists, which further contribute to the scale of CS. So, DID analysis can effectively capture the supply of ecological products in areas where NKEFAs are covered. In general, the enhancement of CS is a gradual and long-term process, and the effect of key ecological function areas and their transfer payments is relatively small in the short term, gradually showing an enhanced trend in the long term. Through continuous vertical ecological compensation for NKEFAs, the supply of ecological products can be continuously improved [7].

### 4.3. Robustness Test

To verify the stability and reliability of the baseline regression results, a series of robustness tests are carried out in this paper.

#### 4.3.1. Placebo Test

**Figure 3:**
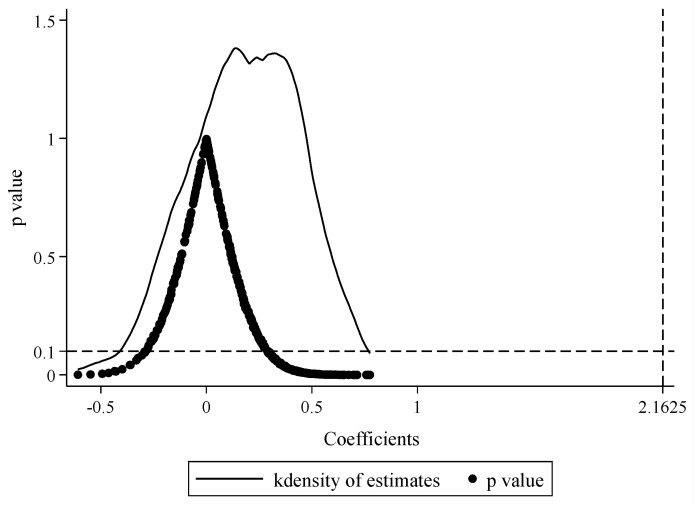
The placebo test of CS. **Note:** The X-axis is the estimated coefficients of DID for the 500 random processes. The curve is the kernel density distribution plot of the estimated coefficients, the dots are the associated *p*-values, and the vertical lines on the right side of the plot are the true estimated coefficients of DID, which are all significantly located in the low-tailed area of the coefficient distribution.

Considering that the changes in CS of the treatment and control groups after the establishment of NKEFAs are also affected by omitted variables, randomness factors, etc., a placebo test is carried out on the baseline regression through a counterfactual framework [42]. The specific operation is to use the non-parametric permutation test method [43,44], with an unduplicated random sample of all prefectures and policy time. Since there are 171 prefectures belonging to the first batch plus the new NKEFAs, we first randomly selected 171 from all prefectures as the treatment group. Then, we selected the rest as the control group and then randomly selected a certain year from 2001 to 2019 as the establishment time of NKEFAs. Finally, we constructed a randomized experiment at two levels of city–year. In order to enhance the explanatory power of the placebo test, the above random process is repeated 500 times, so that the kernel density distribution map of the DID coefficients under 500 random policy shocks can be obtained. If the DID coefficients are no longer significant under random processing and are distributed around 0, it means that the baseline regression results are robust. Figure 3 shows that although the DID coefficient distributions of CS slightly deviated from 0, they are mostly concentrated around 0, and most of the estimated coefficients have *p*-values greater than 0.1. In addition, the estimated coefficient of the baseline regression is located in the low-tailed position of the coefficient distribution, which is clear outlier in the placebo test. The above counterfactual analysis confirms that this paper is not disturbed by omitted variables and random factors in the model setting, and the baseline regression results are robust and reliable.

#### 4.3.2. PSM-DID

The delineation of NKEFAs is not completely random; it is based on the comprehensive assessment of the objectives of building main functional areas and optimizing the territory spatial pattern. Then, it selects the areas that are related to national ecological security, low ecological carrying capacity, etc. [10]; thus, it is prone to endogeneity problems caused by sample selection bias. Propensity Score Matching (PSM) can solve this sample selection problem under the condition of non-randomized experiments [45]. In order to alleviate the bias of sample selection and reduce the estimation bias of DID, the PSM-DID method was further used to evaluate the impact of NKEFAs on CS. Specifically, we use the control variables to predict the probability of each city being designated as a NKEFA (Logit regression), and then, the k-nearest neighbor matching method within the caliper (k taken as 1, i.e., 1:3 matching) is used to match the control group for the sample designated as an NKEFA (treatment group), thus ensuring that there was no significant systematic difference between the treatment group and the control group before the policy shock of NKEFAs. Then, the matched samples are used for DID analysis. The regression result is shown in (1) of Table 3; the estimated coefficient of DID is 2.2004, and it passes the 1% significance level test, which is consistent with the baseline regression, so the positive promotion effect of NKEFAs on CS has robustness.

#### 4.3.3. Excluding Other Policy Interference

When the NKEFAs was established, several other ecological policies to improve the environment were also underway at the same time. Other ecological policies of the same period, such as the Grassland Ecological Protection Subsidy Incentive Policy started in 2011, the New Round of General Program for Returning Cropland to Forests and Grasses in 2014, and the Opinions on Accelerating the Construction of Ecological Civilization in 2015, may also affect the CS and interfere with the policy effect of identifying NKEFAs. In order to exclude the interference of other ecological policies, based on the baseline regression model, we introduced the interaction term of the grouping variable (treat) and the time dummy variables in 2011, 2014, and 2015 [31]. The re-estimated result is shown in (2) of Table 3. It can be found that the estimated coefficient of DID is still significantly positive, and the baseline estimation result is robust. Compared with the baseline regression, the positive effect of NKEFAs on CS is increased after excluding the interference of other ecological policies.

#### 4.3.4. Substituting Explained Variables

Vegetation changes are more sensitive to the improvement of ecological environment. The Normalized Difference Vegetation Index (NDVI) is closely related to vegetation cover, biomass and productivity [6], and the magnitude of NDVI can laterally reflect the ability of a region to provide ecological products and can be used as a proxy variable for CS. The re-estimated result is shown in (3) of Table 3. It can be found that the estimated coefficient of DID on NDVI is significantly positive, so the NKEFAs significantly increase the NDVI level, thus benefiting the supply of ecological product, indicating that the positive contribution of NKEFAs to CS remains robust after substituting the explanatory variables.

#### 4.3.5. Eliminating Special Samples

The establishment of national key ecological function areas may be influenced by factors such as geographical location, endowment conditions, environmental carrying capacity, spatial development pattern, etc. Cities such as province-level municipalities, provincial capitals, and special economic zones may differ from other cities due to their own location conditions and ecological characteristics. We eliminated these samples and retained a total of 292 other city samples for re-estimation; the result is shown in (4) in Table 3. It can be found that the estimated coefficient of DID is still significantly positive, so the positive contribution of NKEFAs to CS remains robust after eliminating some samples.

### 4.4. Mechanism Analysis

According to the previous analysis, the establishment of NKEFAs may affect CS through the optimization of territorial spatial structure (TERRI), industrial structure upgrading (INDUS) and labor transfer flow (LABOR). To verify these mechanisms, it is necessary to define these mediating variables. ① TERRI. The territory can be divided into urban space, agricultural space, ecological space and other space. Ecological space is the space with the main function of providing ecological products or ecological services, mainly forest land, grassland and water area, and it also includes sandy land and saline land, which is the spatial area that NKEFAs focus on [5]. We characterize the TERRI by the proportion of ecological space, i.e., the proportion of the sum of forest land, grassland, water and other ecological space to the territory area. ② INDUS. Industrial structure upgrading mainly refers to the transformation and advanced process of leading industries to industry and services [46]. The ratio of the added value of the tertiary sector to the added value of the secondary sector is used to characterize INDUS and reflect the trend of advanced industrial structure. ③ LABOR. Labor mobility mainly refers to the transfer of labor force in regions and industries. The spatial distribution of counties covered by NKEFAs has a high overlap with that of poverty-stricken counties nationwide [47], which promotes the transfer of some populations to urbanized areas and can trigger the movement of rural labor between industries. We characterize LABOR by the ratio of labor in agriculture, forestry, animal husbandry and fishery in the total population at the end of the year.

We analyzed the impact of NKEFAs on CS by TERRI, INDUS, and LABOR [48] (Table 4). Columns (1), (2), and (3) test the effects of NKEFAs on each of these mediating variables, and columns (2), (4), and (6) test the effects of NKEFAs and these mediating variables on CS [49].

For TERRI: The NKEFAs have a significant positive impact on TERRI; that is, the establishment of NKEFAs helps to expand the ecological space in the covered areas and increase its proportion in the territory. Increasing the proportion of ecological space has a significant positive impact on CS, so the optimization of TERRI significantly improves the CS. The TERRI plays a positive mediating role in the impact of NKEFAs on CS, and NKEFAs can enhance CS by optimizing the spatial structure of the territory.

For INDUS: The NKEFAs have a significant positive impact on INDUS; that is, the establishment of NKEFAs helps to promote the upgrading of industrial structure in the covered areas and increase the proportion of tertiary industry in the national economy. The upgrading of industrial structure has a significant positive contribution to CS; industrial transformation helps to enhance the supply capacity of ecological products. In a word, the INDUS plays a significant positive mediating role in NKEFAs affecting CS, and NKEFAs can enhance CS by industrial structure upgrading.

For LABOR: The NKEFAs have a significant positive impact on LABOR; that is, the establishment of NKEFAs helps to promote inter-industrial flow of labor in the covered areas and increase the proportion of labor in agriculture, forestry, animal husbandry and fisheries. The possible reason is that the NKEFAs encourage the development of industries that are suitable for the positioning of the main functional areas. Although the overloaded population has been transferred in an orderly manner, the active population migration policy has increased the population agglomeration and absorption capacity, while the development of ecological industries has also attracted the return of outgoing labor. Increasing the proportion of labor force in agriculture, forestry, animal husbandry and fishery has a significant positive impact on CS; the structural change of labor force significantly enhances the CS. LABOR plays a positive mediating role in the impact of NKEFAs on CS, and NKEFAs can enhance CS by the inter-industrial mobility of labor.

### 4.5. Heterogeneity Analysis

Due to the heterogeneity of factors such as ecological foundation, endowment conditions, and geographical location, the impact of NKEFAs on CS differs among regions. So, it is necessary to conduct the heterogeneity analysis of the baseline regression results. The heterogeneity analysis will be investigated from the following perspectives: different ecological function types; different geospatial locations, including the six geographic zones and both sides of the Hu Huanyong Line; different quantiles of CS.

#### 4.5.1. Different Ecological Function Types

According to the differences in the various ecological products and services provided by NKEFAs and their functional positioning, they can be classified into four types: water conservation, soil conservation, windbreak sand-fixation, and biodiversity maintenance. Specially, the function areas of water conservation strictly protect the natural vegetation with the water-conserving function; they prohibit overgrazing, disorderly mining, deforestation, grassland reclamation, etc. The function areas of soil conservation vigorously promote water-saving irrigation and rainwater storage utilization, develop dry water-saving agriculture, strengthen the comprehensive management of small watersheds, implement mountain closure and a grazing ban, and then restore degraded vegetation. The function areas of windbreak sand-fixation restore grassland vegetation and strictly control the livestock load by increasing the efforts to return farmland to forest and pasture. The function areas of biodiversity maintenance prohibit the indiscriminate harvesting of wild animals and plants, maintain and restore the balance of wild animal and plant species, and then realize the virtuous cycle and sustainable use of wild animal and plant resources.

Overall, the ecological function areas of water conservation, soil conservation, windbreak sand-fixation and biodiversity maintenance all significantly enhanced CS, but there are differences in the enhancement effect of different functional area types (Table 5). In comparison, the windbreak sand-fixation type of NKEFAs has the strongest enhancement effect on CS, which is followed by soil conservation type, and the biodiversity maintenance type has the weakest enhancement effect on CS.

The windbreak sand-fixation type of NKEFAs is generally located in fragile ecosystems with high desertification and severe grassland degradation, but deserts are an important part of terrestrial ecosystems, which can store a large amount of carbon dioxide and play a key role as carbon sinks [50]. The soil conservation type has the functions of avoiding soil erosion through watershed management, limiting resource development, returning farmland to forest and grass, etc. Water conservation type has the functions of retaining precipitation, regulating runoff, controlling soil desertification, and restoring vegetation, which can effectively promote the benign cycle of ecosystem water. Biodiversity maintenance type has the functions of maintaining and restoring the balance of wildlife species and populations, preventing habitat changes caused by ecological construction, effectively improving the stability of ecosystems, and promoting the diversification of ecological product supply capacity.

#### 4.5.2. Different Geographic Regions

According to the variability of ecological endowment conditions and sensitivity in different geographic spaces, the country is divided into regions using two approaches: the six geographic regions and the two sides of Hu Huanyong Line. The six geographic regions are Northeast China (NEC), North China (NC), Northwest China (NWC), Southwest China (SWC), Middle and Lower Reaches of the Yangtze River regions (MLY) and Southeast Coastal regions (SEC), of which the NEC, NC, and NWC belong to the north, and the SWC, MLY, and SEC belong to the south (Appendix A). The Hu Huanyong Line is the dividing line in China of natural geographic conditions and human geographic differences, and the east–west sides also reveal the regional difference of resource and environmental endowment base.

The results in Table 6 show that the establishment of NKEFAs has enhanced CS in the six geographic regions and on both sides of the Hu Huanyong Line, but the enhancement effect varied from region to region. After comparison, in terms of the six geographic regions, the enhancement effect is shown as NWC > NEC > NC > SWC > SEC > MLY, which shows that the enhancement effect of NKEFAs on CS in the north is higher than that in the south significantly. On the both sides of the Hu Huanyong Line, the enhancement effect is shown as west side > east side, and the NKEFAs on the west side of Huanyong Line are dominated by windbreak sand-fixation type, and the ecosystem is vulnerable, which is closer to the estimation result of this function area type.

The types of NKEFAs in SEC mainly include windbreak sand-fixation, water conservation and soil conservation. The functions of different types are intertwined and can effectively improve their capacity of CS. The NKEFAs in the NEC are dominated by forests and wetlands with a better ecological endowment base, whose main function is water conservation, which is conducive to sequestering carbon dioxide. The NC faces an outstanding contradiction among population, economy and environmental-carrying capacity, but it has a rich ecological endowment base and greater potential of vegetation carbon sink. For the SWC, MLY, and SEC in the south, the types of NKEFAs in these regions are mainly based on soil conservation and biodiversity maintenance. The soil conservation type has the function of effectively avoiding soil erosion, while the biodiversity maintenance type helps balance the ecosystem and effectively sequester carbon dioxide to offset the carbon emissions generated by human activities.

#### 4.5.3. Different Quantiles of CS

**Figure 4:**
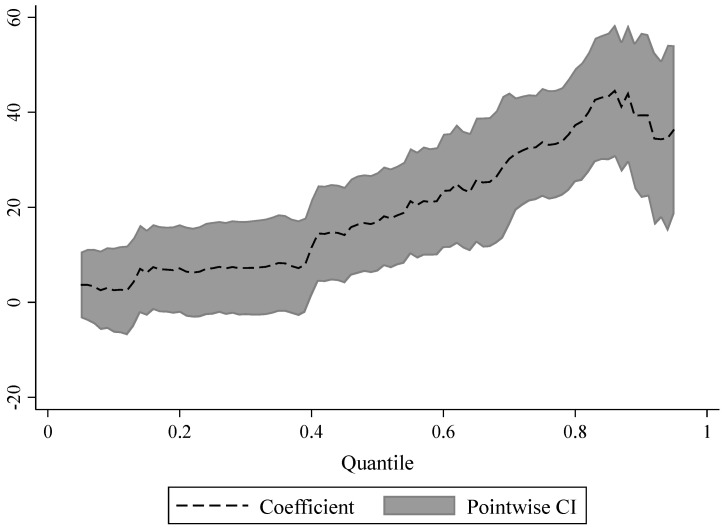
Distribution trend of DID coefficients in different quantiles.

Given the variability in the degree of reliance on ecological policies among regions with different vegetation carbon sink endowment, the distribution heterogeneity of CS in the NKEFAs is examined through a panel quantile regression model (Table 7). The positive effect of NKEFAs on CS shows a rising, then declining, and overall rising trend with the increase in the quantile and produces the highest positive effect near the 85% quantile, indicating that the positive contribution of NKEFAs on CS is higher in areas with a good ecological endowment base (Figure 4). The possible explanation is that areas with a rich ecological base and environmental conditions, which are the key areas restricted and prohibited from development by the state, have a higher environmental carrying capacity themselves and can provide more ecological goods and services, and they also have a larger scale of CS and stronger ability to absorb and store CO_2_. In addition, the establishment of NKEFAs promotes the optimization of territory space and limits large-scale and high-intensity urbanization and industrial development, thereby further releasing the potential room for higher carbon sink.

## 5. Expanded Analysis

### 5.1. Whether the Policy Effect of NEKFAs Has Ecological Spillover to the Neighboring Areas?

In order to investigate whether the enhancement role of CS by the NKEFAs has an ecological spillover effect on the neighboring areas, drawing on the method of existing studies [51,52], it is assumed that in addition to the treatment group, the areas adjacent to the treatment group in the control group are also indirectly affected by this policy, and this sample set can be called the neighboring group (close to treatment group). Then, the neighboring group is introduced into the DID model together with the treatment group. The results are shown in columns (1) and (2) of Table 8.

There is a significant positive spillover effect of NKEFAs on CS in their neighboring areas, indicating that the establishment of NKEFAs not only enhances the CS of the covered area but also enhances the CS of its surrounding neighboring areas through radiation. NKEFAs and their neighboring areas have similar ecological endowment and natural conditions, and the existence of a demonstration effect and warning effect makes neighboring governments compete to imitate and learn from each other under the increasingly strict policy and environmental regulations. Driven by the radiation of NKEFAs, the neighboring areas have issued appropriate ecological policies according to the target decision-making and behavioral bias of local environmental protection [53], thereby helping to improve their own ecological products.

### 5.2. Whether the Ecological Objectives of NEKFAs Be Balanced with Economic Growth?

One of the ecological objectives of NKEFAs is to enhance the supply capacity of ecological products; their industrial policies implement a strict negative list system for industrial access and plan socio-economic development in accordance with the positioning of main functional areas. Although the central government has established a transfer payment policy for NKEFAs, the impact of restricted development on economic growth needs further evaluation. We evaluate the impact of NKEFAs on economic growth from the total and per capita levels and re-estimate the model with the logarithm of GDP (lnGDP) and the logarithm of per capita GDP (lnPGDP) as explained variables. From columns (3) and (4) of Table 8, the NKEFAs have a significant positive impact on regional GDP and GDP per capita. Restricted development does not affect local economic growth but instead promotes economic development, indicating that NKEFAs can promote a win–win situation of ecological environment and economic development and achieve the goals of “lucid waters and lush mountains are invaluable assets” [54]. The main reason why ecological environment and economic growth can achieve synergistic development is that the spatial optimization and development constraints of the national territory make it necessary for industrial development to consider environmental carrying capacity and market capacity. On the one hand, ecological transfer payments can effectively motivate local governments to eliminate backward industries and develop green economy, and on the other hand, the strict industrial access system can help upgrade industries and promote the development of special industries and service industries.

## 6. Conclusions

Based on calculating the annual average level of CS, and taking the establishment of NKEFAs as a quasi-natural experiment, the impact of NKEFAs on CS is evaluated using a time-varying DID model. The establishment of NKEFAs has significantly enhanced the capacity of CS. Compared with non-NKEFAs, NKEFAs make CS in the covered areas enhanced by 2.1625 in the average treatment effect, and they are dynamically persistent in the long term. This conclusion has a high degree of robustness. The NKEFAs can enhance CS through territory spatial structure, industrial structure upgrading and labor transfer mobility. The impact of NKEFAs on CS has a heterogeneity of spatial change in different ecological function types, geospatial locations, and quantile levels, with higher enhancement of CS at windbreak-sand fixation type, northwestern region and high quantiles, respectively. The NKEFAs have a positive ecological spillover effect on neighboring areas, and the ecological objective of NKEFAs can be balanced with local economic growth, thus achieving ecological and economic synergy.

## 7. Policy Implications

Based on the conclusions, the main policy insights we received are the following:

(1) Improve the sustainability of the establishment of NKEFAs. The current system of establishing NKEFAs has generally promoted the realization of enhancing ecological products supply and improving environmental quality, and the enhancement effect has become more and more significant over time. The establishment of NKEFAs has effectively stimulated local governments to act in ecological management and environmental protection. To form a long-term positive incentive and avoid the recurrence of ecological problems in NKEFAs, long-term support and supervision and guidance at the national level are necessary to improve the stability and sustainability of policy implementation.
(2) Build a diversified ecological governance and supervision system for different functional area types and different ecological characteristics. For functional areas with poorer ecological endowments, such as the northwestern region, which is mainly a windbreak sand-fixing ecological functional area, the ecological vulnerability is high, and the overall deterioration of the ecological environment has not been fundamentally curbed, so the ecological management of NKEFAs should continue to be strengthened to improve the overall function of the ecosystem. The central government’s transfer funds for NKEFAs need to be tilted more toward these areas, while local governments should continue to increase investment in environmental protection, strengthen ecological environment supervision, and form a long-term operation mechanism of ecological compensation.
(3) Act strictly in accordance with the requirements of the National Main Function Area Planning. On the one hand, the space for human activities should be controlled beyond the delineated ecological red line and coordinated with the environmental carrying capacity of ecological space; the expansion intensity of production space should also be reasonably controlled to improve the efficiency and sustainability of the territory use. On the other hand, actively developing ecological agriculture and service industries introduces more active population migration policies and household registration management policies, attracts labor to green industries, promotes an efficient market-oriented flow of labor, and accelerates the equalization of basic public services between the floating population and local population.
(4) The existence of spatial spillover effect is hard to ignore. Neighboring ecological function areas should actively explore and build a feasible mechanism for the synergistic linkage of cross-regional cooperation in ecological environment management, industrial green development, and territory spatial utilization. Local governments should abandon the ecological management policies of separate governance and beggar-thy-neighbor, and inter-regional experience learning and joint prevention in ecological policies can effectively promote the achievement of ecological goals and the improvement of management efficiency.

This study inevitably has some limitations. The establishment of NKEFAs is based on the county as a unit, which is limited by the socio-economic data acquisition capacity. This study aggregates the covered counties to their prefecture-level cities according to administrative divisions, and finally, it collects the panel data at the prefecture-level city for estimation, which requires continuous data mining to extensively collect socio-economic data at the county level in the future research. In addition, in contrast to carbon sink, carbon emission is a process, activity or mechanism that releases carbon into the atmosphere. Whether the establishment of NKEFAs can reduce carbon emissions will be explored in depth in the future studies.

## Figures and Tables

**Table 1:** Descriptive statistics of the variables.

Variable	Variable Definition	N	Mean	S.D.	Min.	Max.
Explained variables:						
CS	Vegetation carbon sink scale (million tons)	6270	31.734	35.868	0.061	430.523
Core explanatory variable:						
DID	NKEFAs	6270	0.198	0.398	0	1
Control variables:						
DEN	Total population/land area (people/km^2^)	6270	402.861	485.234	0.656	6729.490
lnPGDP	Logarithm of GDP per capita (2001 as base period)/RMB yuan	6270	10.010	0.937	6.898	12.657
URBAN	Urbanization rate of resident population/%	6270	42.574	19.694	7.435	100
STRUC	Gross secondary industry/GDP	6270	0.384	0.094	0.086	0.835
GAP	Urban per capita disposable income/rural per capita net income	6270	2.685	0.784	0.917	7.378
OPEN	Total import and export trade/GDP	6270	0.187	0.375	0	6.966
TRANS	Road mileage/land area (km/km^2^)	6270	0.801	0.559	0.009	5.887
PRE	Average annual precipitation/mm	6270	954.358	539.161	29.289	2680.360
TEM	Average annual temperature/°C	6270	13.883	5.451	−2.908	25.636
SUN	Sunshine hours/h	6270	2068.952	511.408	784.640	3407.62

**Table 2:** Results of baseline regression.

Variables	Explained Variable: CS			
	(1)	(2)	(3)	(4)
DID	3.5388 *** (0.1333)	2.3985 *** (0.1683)	2.1615 *** (0.1678)	2.1625 *** (0.1743)
DEN	—	—	−0.0016 *** (0.0006)	−0.0011 * (0.0005)
lnPGDP	—	—	1.4101 *** (0.1239)	1.2165 *** (0.2812)
URBAN	—	—	−0.0116 * (0.0064)	−0.0110 * (0.0064)
STRUC	—	—	0.7183 (0.7941)	−0.4380 (0.9840)
GAP	—	—	0.0328 (0.1280)	−0.0780 (0.1319)
OPEN	—	—	−0.5821 ** (0.2889)	−0.4847 * (0.2906)
TRANS	—	—	−0.5008 ** (0.2069)	−0.4486 ** (0.2149)
PRE	—	—	0.0007 *** (0.0002)	0.0006 *** (0.0002)
TEM	—	—	−0.0327(0.0764)	−0.2245 ** (0.0871)
SUN	—	—	−0.0004 * (0.0002)	−0.0005 * (0.0003)
Constant	31.6003 *** (1.7518)	28.6806 *** (0.1896)	19.6504 *** (2.3671)	25.4192 *** (2.9588)
Adj-*R^2^*	0.1062	0.1582	0.1424	0.1688
city FE	NO	YES	NO	YES
year FE	NO	YES	NO	YES
No. of cities	330	330	330	330

Note: *** *p* < 0.01. ** *p* < 0.05. * *p* < 0.10. Robust standard errors clustered at city level appear in parentheses.

**Table 3:** Results of robustness tests.

Variables	PSM-DID	Excluding Other Policy Interference	Substituting Explained Variables	Eliminating Special Samples
	CS	CS	NDVI	CS
	(1)	(2)	(3)	(4)
DID	2.2004 *** (0.0043)	2.1679 *** (0.1781)	0.0185 *** (0.0011)	1.9909 *** (0.1895)
Constant	27.1356 *** (3.2617)	25.4126 *** (2.9581)	0.6039 *** (0.0195)	21.2040 *** (3.1719)
Controls	Yes	Yes	Yes	Yes
Adj-*R*^2^	0.1757	0.1696	0.4931	0.1692
city FE	Yes	Yes	Yes	Yes
year FE	Yes	Yes	Yes	Yes
No. of cities	330	330	330	292

**Note:** *** *p* < 0.01. Robust standard errors clustered at city level appear in parentheses. The nearest neighbor matching adopts a 1:3 matching method; samples that do not satisfy the common support hypothesis are removed after matching. NDVI is obtained from the Chinese annual vegetation index (NDVI) spatial distribution dataset of the Data Center for Resource and Environmental Sciences, Chinese Academy of Sciences, with a spatial resolution of 1 km.

**Table 4:** The results of mechanism test.

Variables	TERRI		INDUS		LABOR	
	TERRI	CS	INDUS	CS	LABOR	CS
	(1)	(2)	(3)	(4)	(5)	(6)
DID	0.0025 *** (0.0003)	2.0658 *** (0.1753)	0.0121 * (0.0065)	2.1437 *** (0.1737)	0.0070 *** (0.0013)	2.1128 *** (0.1745)
TERRI		38.1633 *** (8.4921)				
INDUS				1.5487 *** (0.2341)		
LABOR						7.0843 *** (1.7342)
Constant	0.4885 *** (0.0045)	6.7750 (5.0929)	0.3239 ** (0.1638)	24.9176 *** (2.9491)	0.2968 *** (0.0222)	23.3168 *** (2.9993)
Controls	Yes	Yes	Yes	Yes	Yes	Yes
Adj-*R^2^*	0.3338	0.1716	0.6547	0.3345	0.3305	0.1711
city FE	Yes	Yes	Yes	Yes	Yes	Yes
year FE	Yes	Yes	Yes	Yes	Yes	Yes
No. of cities	330	330	330	330	330	330

Note: *** *p* < 0.01. ** *p* < 0.05. * *p* < 0.10. Robust standard errors clustered at city level appear in parentheses.

**Table 5:** Heterogeneity analysis: differences based on function type.

Variables	Water Conservation	Soil Conservation	Windbreak Sand-Fixation	Biodiversity Maintenance
	(1)	(2)	(3)	(4)
DID	1.1967 *** (0.2137)	2.0411 *** (0.2815)	7.7708 *** (0.3853)	0.6964 *** (0.2543)
Constant	22.4709 *** (2.9962)	26.7325 *** (3.0078)	25.1438 *** (2.8970)	24.2242 *** (2.9958)
Controls	Yes	Yes	Yes	Yes
Adj-*R^2^*	0.1516	0.1546	0.2020	0.1482
city FE	Yes	Yes	Yes	Yes
year FE	Yes	Yes	Yes	Yes
No. of NKEFAs	95	40	18	52
No. of cities	330	330	330	330

Note: *** *p* < 0.01. Robust standard errors clustered at the city level appear in parentheses. The sum of functional areas number included in each type is not equal to 171, because some cities belong to two types.

**Table 6:** Heterogeneity analysis: regional differences based on geographic space.

Variables	NEC	NC	NWC	SWC	MLY	SEC	West side	East side
	(1)	(2)	(3)	(4)	(5)	(6)	(7)	(8)
DID	2.4679 *** (0.4665)	2.0233 *** (0.2149)	3.7120 *** (0.7461)	0.7847 * (0.4041)	0.5737 *** (0.1501)	0.6091 *** (0.2186)	3.0197 *** (0.9017)	1.7315 *** (0.1332)
Constant	41.7258 *** (9.8409)	26.0882 *** (4.9821)	16.5328 *** (10.7054)	53.7831 *** (8.1748)	28.3889 *** (3.5909)	38.7921 *** (4.5361)	13.9183 *** (11.7277)	29.7897 *** (2.3952)
Controls	Yes	Yes	Yes	Yes	Yes	Yes	Yes	Yes
Adj-*R*^2^	0.3559	0.5711	0.2611	0.3891	0.3576	0.2888	0.1944	0.2338
city FE	Yes	Yes	Yes	Yes	Yes	Yes	Yes	Yes
year FE	Yes	Yes	Yes	Yes	Yes	Yes	Yes	Yes
No. of cities	36	59	63	47	67	58	57	273

Note: *** *p* < 0.01. * *p* < 0.10. Robust standard errors clustered at city level appear in parentheses. The six geographic regions include the following provinces: Heilongjiang, Jilin and Liaoning in NEC, Beijing, Tianjin, Hebei, Henan, Shandong and Shanxi in NC, Inner Mongolia, Shaanxi, Gansu, Ningxia, Qinghai and Xinjiang in the NEC, Sichuan, Chongqing, Guizhou, Yunnan and Tibet in the SWC, Jiangsu, Shanghai, Anhui, Jiangxi, Hubei and Hunan in MLY, and Zhejiang, Fujian, Guangdong, Guangxi, Hainan and Hong Kong, Macao and Taiwan in SWC. There are 273 cities on the east side of the Hu Huanyong Line and 57 cities on the west side.

**Table 7:** Heterogeneity analysis: differences in distribution based on quantile levels.

Quantile		DID	Controls	City FE	Year FE	No. of Cities
(1)	0.05	4.2911 *** (0.1783)	Yes	Yes	Yes	330
(2)	0.25	6.0542 *** (0.1052)	Yes	Yes	Yes	330
(3)	0.50	17.4855 *** (0.2128)	Yes	Yes	Yes	330
(4)	0.75	35.0966 *** (0.2275)	Yes	Yes	Yes	330
(5)	0.95	16.5249 *** (1.1962)	Yes	Yes	Yes	330

Note: *** *p* < 0.01. Robust standard errors clustered at city level appear in parentheses. The coefficient covariance estimation is obtained by bootstrap 1000 times, and the optimization technique selects the adaptive MCMC method.

**Table 8:** Results of expandability analysis.

Variables	Ecological Spillover Effect		Economic Growth	
	CS	CS	lnGDP	lnPGDP
	(1)	(2)	(3)	(4)
DID	2.6682 *** (0.4691)	2.4188 *** (0.4395)	0.0299 *** (0.0082)	0.0491 *** (0.0084)
Spillover effect	0.5395 *** (0.1644)	0.4858 *** (0.1656)		
Constant	28.6401 *** (0.3257)	22.7182 *** (5.2892)	5.7650 *** (0.0704)	9.5025 *** (0.0712)
Controls	No	Yes	Yes	Yes
Adj-*R^2^*	0.1606	0.1700	0.0299	0.9498
city FE	Yes	Yes	Yes	Yes
year FE	Yes	Yes	Yes	Yes
No. of cities	330	330	330	330

Note: *** *p* < 0.01. Robust standard errors clustered at city level appear in parentheses.

**Figure A1:**
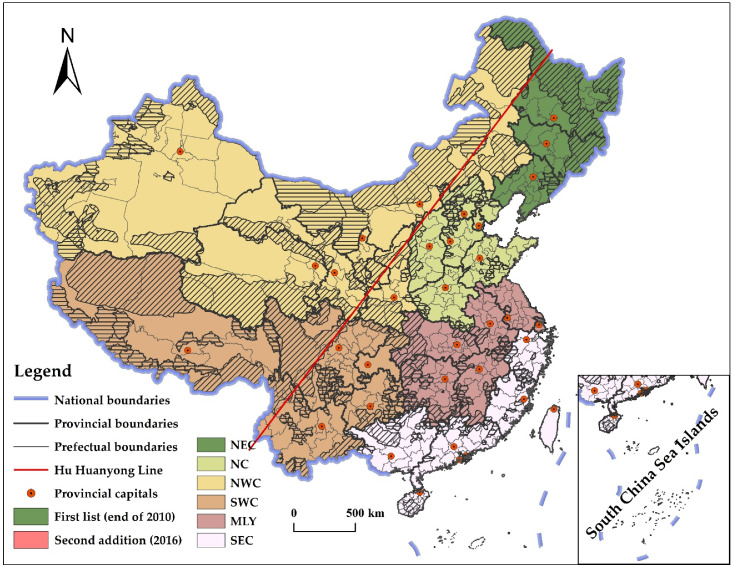
Distribution of NKEFAs and six geographic regions.

## Data Availability

The data presented in this study are available on request from the corresponding author. The data are not publicly available due to data management.
